# Of Beavers and Tables: The Role of Animacy in the Processing of Grammatical Gender Within a Picture-Word Interference Task

**DOI:** 10.3389/fpsyg.2021.661175

**Published:** 2021-07-08

**Authors:** Ana Rita Sá-Leite, Juan Haro, Montserrat Comesaña, Isabel Fraga

**Affiliations:** ^1^Cognitive Processes and Behaviour Research Group, Department of Social Psychology, Basic Psychology, and Methodology, University of Santiago de Compostela, Santiago de Compostela, Spain; ^2^Department of Psychology, Research Center for Behavior Assessment (CRAMC), Rovira i Virgili University, Tarragona, Spain; ^3^Research Unit in Human Cognition, Centro de Investigação em Psicologia (CIPsi), School of Psychology, University of Minho, Braga, Portugal; ^4^Center for Cognitive Science (C3), Nebrija University, Madrid, Spain

**Keywords:** animacy, animate monitoring hypothesis, gender acquisition and processing hypothesis, gender congruency effect, grammatical gender, picture-word interference paradigm, semantic prioritization

## Abstract

Grammatical gender processing during language production has classically been studied using the so-called picture-word interference (PWI) task. In this procedure, participants are presented with pictures they must name using target nouns while ignoring superimposed written distractor nouns. Variations in response times are expected depending on the congruency between the gender values of targets and distractors. However, there have been disparate results in terms of the mandatory character of an agreement context to observe competitive gender effects and the interpretation of the direction of these effects in Romance languages, this probably due to uncontrolled variables such as animacy. In the present study, we conducted two PWI experiments with European Portuguese speakers who were asked to produce bare nouns. The percentage of animate targets within the list was manipulated: 0, 25, 50, and 100%. A gender congruency effect was found restricted to the 0% list (all targets were inanimate). Results support the selection of gender in transparent languages in the absence of an agreement context, as predicted by the Gender Acquisition and Processing (GAP) hypothesis (Sá-Leite et al., 2019), and are interpreted through the attentional mechanisms involved in the PWI paradigm, in which the processing of animate targets would be favored to the detriment of distractors due to biological relevance and semantic prioritization.

## Introduction

In European Portuguese (EP), the nouns “*mesa*” (table) and “*castor*” (beaver) share a characteristic that is not present in other nouns such as “*gato*” (cat): the first two have grammatical gender, but the latter has what is called natural gender. Similar to other gendered languages, EP has a gender system that classifies nouns according to different values or classes (Corbett, 1994). More specifically, in most Romance languages, nouns can be either feminine (e.g., “*mesa*” [table] or “*gata*” [female cat]) or masculine (e.g., “*relógio*” [watch] or “*gato*” [male cat]). However, those values hold different representational implications depending on the gender types mentioned above (Caramazza, 1997). Whereas grammatical gender is purely abstract (“*mesa*” and “*relógio*”), natural gender has a basic semantic relationship with the meaning of the noun in question (the biological sex marked through gender morphemes, “*gat****a***” and “*gat****o***,” see Corbett, 1991; Johnson, 2014). This distinction is closely linked to animacy, which has been shown to be a determining factor in the establishment of many regularities and cut-off points related to grammar and syntactic function (Dahl, 2008). Animacy is often described in terms of a hierarchy reflected in what is quite a flexible continuum, ranging from inanimate things to living entities (things < animals < humans; Silverstein, 1986; Dahl, 2000). Our linguistic system seems to hold a bias to assign animates to syntactically prominent positions (Branigan et al., 2008), showing a default preference for active sentences over passive ones, definiteness over indefiniteness, subject over non-subject elements, among many more. In fact, animates can even revert well-stablished syntactic tendencies, such as those in Spanish or EP to attach themselves high up inside the complex noun phrases in relative clauses (Acuña et al., 2009; Soares et al., 2010). Regarding gender, whereas inanimate nouns have grammatical gender, animate nouns may have either grammatical or natural gender. Hence, grammatical gender encompasses nouns across the complete hierarchical continuum of animacy, but natural gender is restricted to animate nouns. In this sense, nouns with grammatical gender such as inanimates “*mesa*” and “*relógio*,” or animates “*castor*,” “*macaco*” (beaver, monkey, both masculine [M]) and “*cegonha*,” “*zebra*” (stork, zebra, both feminine [F]) take an inherent gender value which is immovable and cannot be replaced. This intrinsic gender (Corrêa et al., 2011) is often described as an arbitrary lexical-syntactic characteristic of nouns (Schriefers and Jescheniak, 1999), since it has no semantic or form implications for nouns themselves. However, for the sake of agreement, it is reflected in the form of certain words contained in speech (e.g., “*a* *mesa bonit**a*,” the beautiful table; but “*o* *castor bonit**o*,” the beautiful beaver). On the other hand, for animate nouns with natural gender, these gender values are optional. Instead of being inherently classified as masculine or feminine, the biological sex of the referent occupies a central role for gender assignment, and hence these nouns can take different values depending on whether the referent is perceived as male or female. By virtue of the gender value they adopt, they modify their own form via gender morphemes (e.g., “*o* *gat**o*” [the male cat] and “*a* *gat**a*” [the female cat])^1^.

In the present study, we are interested in the representation and processing of grammatical gender in language production as an inherent and purely abstract characteristic of both inanimate and animate nouns. Over the last three decades, research on gender selection during lexical access has remained controversial due to the lack of conclusive results. Essential questions are still unclear: Is an agreement context necessary for the gender of a noun to be processed (producing “*a mesa bonita*” vs. “*mesa*”)? Are there other requirements for gender processing in language production? Do the answers to such questions depend on the language itself? Why do Romance and Germanic language families seem to behave differently? One of the variables that may be relevant to debates in this area is animacy. Yet the animacy status of the experimental stimuli of all studies in this area has been systematically ignored, not only in the design of experiments, but also indirectly when it comes to the experimental control of the materials. This is surprising, because: (1) the core of gender systems is closely linked to animacy (Corbett, 1991; Dahl, 2000); (2) it has been suggested that this link is especially relevant in languages with dual-gender systems (i.e., Romance languages; Sedlmeier et al., 2016); (3) our linguistic system seems to hold a bias to assign animates to syntactically prominent positions, as previously noted (Branigan et al., 2008); and (4) the animacy of stimuli (words and images) has shown consistent cognitive repercussions in our perception of the surroundings and our response to the world (New et al., 2007, 2010). Thus, bearing in mind that grammatical gender includes both animate and inanimate nouns, it seems crucial to consider animacy as a significant factor in the study of grammatical gender representation and processing. In what follows, we will address the theoretical and experimental aspects of research on grammatical gender, highlighting the possible cognitive and linguistic repercussions of including animate nouns in the design of an experiment along with inanimate ones. Two experiments in which the percentage of animate targets was manipulated (0, 25, 50, and 100%) were conducted to examine the role of this variable in gender processing. Overall, only the list without animate targets showed gender effects, and hence such findings underline the importance of animacy in gender studies, illustrating what many researchers have theoretically described: the superiority and specificity of animacy on language processing and cognition.

### Theoretical and Experimental Aspects of Grammatical Gender Processing

The representation and processing of grammatical gender during the spoken production of nouns has been typically studied through the so-called picture-word interference (PWI) paradigm (Rosinski et al., 1975). In this Stroop-like task, participants see pictures that they are asked to name aloud using a target noun while ignoring superimposed distractor nouns presented at the same time or in temporal proximity with the pictures. By manipulating the gender values of the target and the distractor nouns, researchers create the main conditions of gender congruency and incongruency (e.g., gender congruent condition: target “*mesa*” [F, table] and distractor “*flor*” [F, flower]; gender incongruent condition: the same target but paired with distractor “*relógio*” [M, watch]). As both target and distractor nouns have been shown to compete for selection (as evidenced from the semantic relatedness effect, see Schriefers and Jescheniak, 1999), variations in the response times (RTs) depending on their gender congruency status are expected.

Briefly, results described in the literature show that for native speakers of Germanic and Slavic languages (namely Dutch, German, and Czech) faster naming responses to the gender congruent condition than to the gender incongruent condition are systematically observed when participants use noun phrases to name the pictures (gender congruency effect, GCE). Yet, this only occurs if each gender value has its own different definite article, e.g., in German, “*der*” (M), “*die*” (F), and “*das*” (Neuter), in other words, only if gender is necessary to determine the form of other elements for the sake of agreement. Null results are obtained with bare nouns or with noun phrases in which the definite article has the same form for all genders (e.g., in German, plural definite article for all values is “die”; Schriefers, 1993; Van Berkum, 1997; La Heij et al., 1998; Schriefers and Teruel, 2000; Schiller and Caramazza, 2003; Bordag and Pechmann, 2008).

However, studies testing native speakers of Romance languages reveal a more complex situation, drawing attention to the fact that gender representation and processing may be closely tied to the characteristics of languages themselves (see Sá-Leite et al., 2019). More specifically, it seems that gender competitive effects can be obtained even without a context of agreement, which has been pointed out as mandatory for the processing of gender in Germanic and Slavic languages (see the WEAVER++ model, Levelt et al., 1999). Whereas certain studies featuring bare nouns show null effects with native speakers of Spanish, Italian, and French (see Finocchiaro et al., 2011), others consistently find gender effects. For instance, Cubelli et al. (2005) and Paolieri et al. (2010a, 2011) tested native speakers of Italian and Spanish and obtained the reverse pattern of a GCE, that is, a gender incongruency effect (GIE).

Two theoretical approaches have been advanced as possible explanations of this cross-linguistic discrepancy: the Double-Selection Model (Cubelli et al., 2005) and the Gender Acquisition and Processing (GAP) hypothesis (Sá-Leite et al., 2019). Both proposals focus on the concept of phonological gender transparency (Bates et al., 1995). In most Romance languages, including Italian, EP, and Spanish, we find simple and extended statistical regularities in the nominal endings that are associated with each gender value. Thus, while transparent nouns display these regularities (i.e., in EP transparent nouns end in “-a” for feminine [“*mesa*”] and “-o” for masculine [“*relógio*”]), opaque nouns do not (e.g., in EP nouns that end in other letters such as “-e,” “*torre*” [tower, feminine] and “*pente*” [comb, masculine]). The Double-Selection Model by Cubelli et al. (2005) proposes a structure of lexical access in which the flow of information goes through two levels of representation: a conceptual-syntactic stratum and a morpho-phonological stratum; grammatical gender being located at the first level, which supposedly integrates both semantic and grammatical information. Therefore, gender values would be characteristics inherently describing each abstract word representation (i.e., each lemma) along with other semantic and grammatical features. Critically, the authors propose that gender must be processed in transparent languages because it is necessary to encode the nominal endings later in the morpho-phonological strata (“-a” for feminine, “-o” for masculine). Since gender has to be processed, Cubelli et al. argue that competition occurs through similarity. The activation of a lemma node would activate other lemma nodes with similar semantic and grammatical features, and thus similar lemmas would compete for selection. If target and distractor nouns are both feminine, they are more similar and hence competition for lemma selection increases. This translates into a GIE. Conversely, the nouns in languages such as Dutch and German do not fall within the definition of transparency, and thus gender does not have to be processed for the sake of a noun's form, the results therefore being null when bare nouns are produced. The model also accounts for the GCE observed with noun phrases in Germanic and Slavic languages: in those languages, although nouns do not have nominal endings to be processed, when agreement has to be fulfilled, nouns of the same gender will automatically send activation to the same determiner form, facilitating determiner selection and reducing reaction times (RTs) in the gender-congruent conditions. However, this proposal has raised criticism. On the one hand, the conception of gender as a quasi-semantic feature in a common conceptual-grammatical lemma level seems incompatible with results from other types of studies on grammatical gender, especially those concerning the cross-linguistic gender congruency effect in bilinguals, in which nouns of the same gender seem to facilitate lexical access rather than hampering it (e.g., Paolieri et al., 2010b; Morales et al., 2011; Manolescu and Jarema, 2015). On the other hand, the authors equate phonological gender regularities (“-a” in “*mesa*”) to gender morphemes. Gender morphemes, however, are restricted to nouns with natural gender (“*gat-o*,” “*gat-a*”). The idea that gender must be processed to encode the ending of a noun with abstract grammatical gender, such as “*mesa*” in which the “-a” is a mere thematic vowel, is controversial; yet more so if we consider that opaque nouns have also been shown to entail gender effects (Cubelli et al., 2005; Paolieri et al., 2010a, 2011) and that these have final letters that do not correlate with any gender value (to better assess these criticisms, see Finocchiaro et al., 2011).

**Figure 1:**
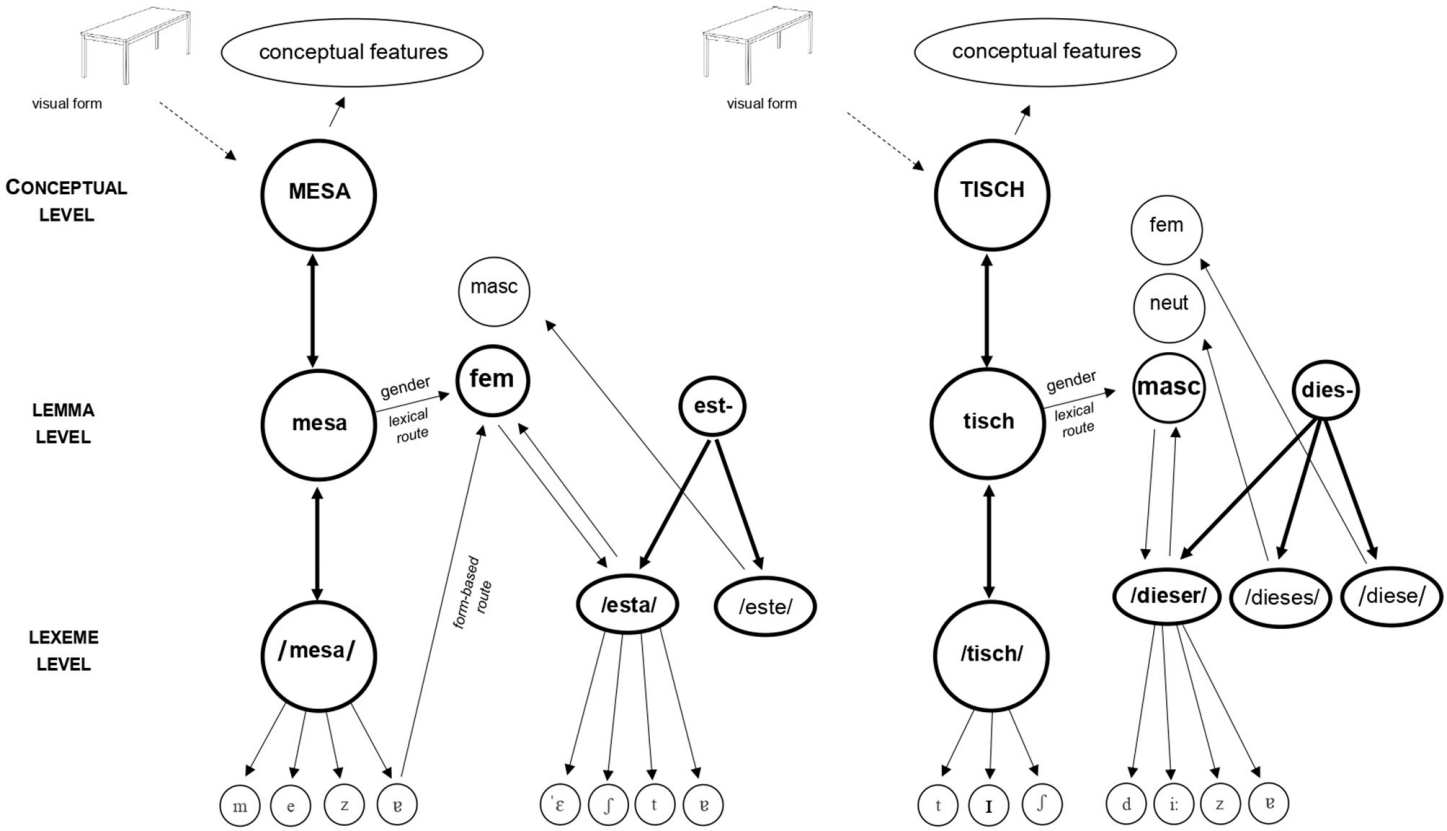
Lexical network (simplified) proposed by the GAP hypothesis. Representation of the spoken production of the noun phrase “this table” in Portuguese “*esta mesa*” (feminine; on the left) and in German “*dieser Tisch*” (nominative case, masculine; on the right). Gender values are represented through gender nodes that accumulate activation. Bidirectional connections and cascaded flow of information is conceived as in Dell's model. The form-based and lexical routes from the Dual Route model of gender retrieval (Gollan and Frost, 2001) of language comprehension are adapted to language production (Sá-Leite et al., 2021). For more information on the differential processing of transparent and opaque nouns, see Sá-Leite et al. (2021).

Alternatively, the GAP hypothesis is based on the commonly accepted structure of lexical access (Levelt, 1989, 1992; Roelofs, 1992). It conceives a first level of conceptual encoding, a second level of syntactic and grammatical encoding—namely the lemma, and a final level of morpho-phonological encoding—namely the lexeme, aside from additional mechanisms of motor articulation (see Figure 1). Like classical models of language production, such as the WEAVER++ proposal (Levelt et al., 1999) or the Independent Network model (Caramazza, 1997), it locates grammatical gender in the syntactic-grammatical (lemma) stratum. There, gender values are represented via gender nodes (e.g., in the mind of a Portuguese speaker, there would be a masculine node and a feminine node) to which all nouns are connected unidirectionally depending on their value. These gender nodes accumulate activation coming from the words' lemmas and compete for selection, predicting gender congruency rather than incongruency effects. Besides, the model embraces the interactive principles of lexical access (see Dell, 1986, 1990) and so the connections between the three encoding levels are bidirectional and the flow of activation is cascaded. Following this conception of lexical access, if a native speaker of German sees the picture of a “table,” the concept of TABLE would be activated in the conceptual level, which would send activation to the lemma “Tisch” at the syntactic-grammatical stratum. The lemma “Tisch” would then send activation to the masculine gender node. A flow of activation would also be occurring from the lemma to the lexeme level in which the morpho-phonological representation of the word (/tisch/) would be encoded. Importantly, the main postulate of this model states that there are differences in the basal level of activation of the connections between nouns and gender nodes as well as in the gender nodes themselves depending on the general level of gender transparency of the languages. More specifically, many Germanic languages have been identified as opaque for gender, but most Romance languages fall on the transparent side, and hence gender is extremely visible in the form of the words, especially with regard to nouns themselves (Velnić, 2020). In languages such as German and Dutch, the gender system has been described as highly complex in terms of gendered morphophonology, in the sense that even though gender can be predicted for many nouns, the number of regularities is so high and the exceptions are so many that it is not clear to what extent these are cognitively useful (in fact, relative and even absolute complexity in the gender assignment system has been linked to a slower speed of gender acquisition; Audring, 2017, 2019). For example, in German there are more than 40 regularities associated with gender in mono-morphemic nouns (Köpcke, 1982; Köpcke and Zubin, 1983), there are dozens of nominal endings correlating with different gender cues, and many exceptions and overlaps depending on the plurality and case of the nouns involved (Köpcke, 1982). These regularities have shown to be sometimes useful for gender categorization and even comprehension under certain task circumstances, but there is no evidence of their usefulness for language production (e.g., Schiller et al., 2003; Hohlfeld, 2006). Similar conclusions can be drawn for Dutch, often described as one of the most opaque languages when it comes to gender, in its case due to the absence of gender regularities, which increases the covertness of the gender assignment system and thus its complexity (Unsworth, 2013; Audring, 2017). However, EP partially fits the definition given by Corbett (1991, p. 118–119) of what would constitute a truly overt gender system in terms of the formal regularities correlating with gender: one in which, ideally, masculine nouns end in “-o” and feminine nouns in “-a.” Those are exactly the gender transparent cues present in Portuguese nouns and alliteratively across all agreeing elements. The substantial presence of simple phonological cues for gender in nominal endings in transparent languages (~70% of nouns are transparent [“-o” and “-a”] in EP, with similar or greater percentages for languages such as Italian and Spanish; Harris, 1991; Bates et al., 1995; Soares et al., 2018), makes understandable why in these cases gender acquisition is mostly driven by the form of nouns (e.g., Pérez-Pereira, 1991; Barreña, 1997; Corrêa et al., 2011; Rodina and Westergaard, 2013). In contrast, in opaque languages, definite articles are the most reliable and regular cues for acquiring gender with no other similar competitor (see Arnon and Ramscar, 2012). Since most encounters with nouns easily trigger gender processing in transparent languages, the GAP hypothesis states that a link between nouns as a grammatical class and gender is constantly fed (for opaque languages, this link would be especially strong for definite articles). Furthermore, this constant encountering with transparent nouns would be responsible for a higher degree of basal activation of the gender nodes. This would explain why gender effects can be obtained with bare nouns regardless of the presence of a phonological gender cue (gender values can be retrieved for both transparent and opaque nouns) and an agreement context (Paolieri et al., 2010a, 2011; for more details on this hypothesis, see Sá-Leite et al., 2021).

Although this proposal seems to provide a better explanation of the processing of gender during the lexical access of bare nouns (without the involvement of a context for agreement) and to fit previous literature on the many research areas of gender (e.g., Paolieri et al., 2010b), it does not explain why Cubelli et al. (2005) and Paolieri et al. (2010a, 2011) found incongruent rather than congruent gender effects with native speakers of Italian and Spanish. Therefore, more research is necessary to understand to what extent their findings and their explanation based on the double selection mechanism are generalizable, especially considering these and other transparent languages. The examination of the impact of variables such as animacy in gender processing might also shed light on the debate, as we noted above and will describe in detail in the next section.

### The Role of Animacy in the Picture-Word Interference (PWI) Paradigm and Gender Processing

When conducting a PWI task, the inclusion of animate nouns in the stimuli list might have significant implications on the outcome of the experiments, and even on the predictions of those models mentioned in the previous section. Research in cognitive psychology exploring human memory, perception, visual attention, and language processing has shown that our mind exhibits a clear preference for animate over inanimate stimuli (the so-called animacy effect, see Félix et al., 2019, for a recent overview). For instance: (a) animate words are better recalled than inanimate words (e.g., Bonin et al., 2014; VanArsdall et al., 2014; Leding, 2018; Félix et al., 2019); (b) there is a bias for animate stimuli when deciding where to direct our visual attention, regardless of the degree of the perceived threat (Lipp et al., 2004; New et al., 2010; Altman et al., 2016); (c) some grammatical effects are stronger when animate nouns are at issue (Dank et al., 2015); (d) in lexical decision tasks, legal non-words perceived as referring to animate entities yield faster decision times than those perceived as inanimate (Bonin et al., 2019)^2^; and (e) animate words seem to be acquired faster and more accurately than inanimate ones (Corrêa and Name, 2003; Corrêa et al., 2011). Animate stimuli, then, have a special place in our mind, being prioritized across all parts of cognition.

From a psycholinguistic point of view, animacy is considered to be one of the basic features of semantics, and has been attributed a special processing advantage, at least at the level of conceptual encoding during lexical access, as shown in many semantic categorization and violation studies (Radanović et al., 2016; Xiao et al., 2016; Bonin et al., 2019). In this sense, animate nouns tend to be semantically richer than inanimates, bearing more semantical features in their representations, and inducing a “deeper” conceptual processing of words, which consumes more resources and implies greater processing costs when semantic violations have to be detected (Szewczyk and Schriefers, 2011; Xiao et al., 2016). In terms of gender, it has been shown for language learners that gender agreement with animate nouns is cognitively more demanding than with inanimate nouns (Sagarra and Herschensohn, 2011). Thus, we might think that this deep semantic processing of animates during lexical access could have repercussions at other levels of word processing, namely the lemma, in which grammatical gender is selected. In this sense, animacy might be a reason for skipping gender selection during the processing of bare nouns. So, if animacy is a factor that encourages and enriches conceptual processing due to evolutionary pressures (New et al., 2007; Branigan et al., 2008), a direct connection between the conceptual and morpho-phonological strata, might be preferred, and hence gender processing would be skipped if agreement is not required. This is not an unheard idea, as was already proposed by Caramazza and Miozzo in their Independent Network (IN) model (1997). According to this model, the features belonging to the grammatical-syntactic stratum are only activated/retrieved when required and so lexical access can occur through direct connections between the conceptual and the morpho-phonological levels without the grammatical-syntactic stratum intervening. In the case of animacy, the skipping of gender would allow the system to avoid unnecessary costs and prioritize the production of animate nouns.

From the perspective of other areas of cognitive psychology, the selective nature of animacy has been the basis of a fundamental theory of human attention supported by vast experimental evidence: The Animate Monitoring hypothesis (New et al., 2007). Animates, it is claimed, possess an attentional advantage driven by ontogenetic factors that explains why participants are consistently faster and more accurate at detecting changes in animals (human and non-human) relative to changes in inanimate objects, even when these may constitute a threat for survival (e.g., vehicles). This attentional advantage entails a disadvantage for inanimate stimuli since their detection is distracted by the presence of animates (Altman et al., 2016). In fact, animate entities displayed in images have been shown to recruit visual attention in a way that is highly independent from the context.

In light of these findings from different areas of human cognition, it is not difficult to imagine the implications that animacy might have for a PWI task. On the one hand, the prioritization of conceptual encoding in animates may suppose the skipping of some grammatical encoding characteristics as structurally supported by the IN model. If grammatical gender processing is bypassed for either a target or a distractor noun due to their animacy status, it would not be possible to observe gender effects, since there would be no competition between gender nodes. On the other hand, following the attentional and perceptive repercussions of animacy, the degree of attention given to a target and a distractor may be highly disproportional if one of them is animate. In the case of animate targets, their supposedly high independence from the context may hamper the detection and processing of any distractor. Even if detected, the activation reached by the target noun may be notably higher than that reached by the distractor word, which might then be insufficient to generate any kind of competition. At the same time, animate distractors paired with inanimate targets may also be a source of disruption in the reliability of the results. This is so because any interfering effect may be substantially greater if the degree of activation reached by the distractor is “abnormally” high. However, if both targets and distractors are animate, gender effects might also be stronger, since competitive effects would imply higher levels of activation for both targets and distractors.

Despite this, on reviewing the literature on grammatical gender representation and processing during language production, it becomes clear that the role of animacy has been greatly neglected, which in turn might call into question the rationales that have been developed over years regarding gender effects. More specifically, it is a common practice in such studies to include random animate nouns in the stimuli list (targets and distractors) of a PWI paradigm in a non-proportional way across conditions. Only a few works have included zero animate nouns. In these, a GCE was observed in French with a Stimuli Onset Asynchrony (SOA) of +200 ms (Foucart et al., 2010) but not with a SOA of 0, either in French or in Hebrew (Alario and Caramazza, 2002; Dank and Deutsch, 2015). Yet the majority of studies on this matter include 8 to 33% of animate targets, and 3 to 24% of animate distractors (Schriefers, 1993; Van Berkum, 1997; La Heij et al., 1998; Schriefers and Teruel, 2000; Costa et al., 2003; Schiller and Caramazza, 2003; Cubelli et al., 2005; O'Rourke, 2007; Paolieri et al., 2010a, 2011; Finocchiaro et al., 2011; Finocchiaro, 2013; but no information is provided on animacy or available stimuli is provided in the Appendices of Costa et al., 1999; Miozzo and Caramazza, 1999; Miozzo et al., 2002). The percentages of animate stimuli are especially high in Cubelli et al. (2005) and Paolieri et al. (2010a, 2011) studies, which encounter an unexpected GIE (up to 33.33% of targets and more than 20% of distractors; Cubelli et al., 2005; Paolieri et al., 2010a, 2011)^3^. Inconsistent results associated with these studies may be explained, at least partially, by this. If animacy does have a role in gender processing, future research should more effectively assess or control this variable and revise previous findings.

## The Present Study

In this study, we aimed to explore the representation and processing of grammatical gender in a transparent Romance language that has received less attention, EP, while assessing the impact of animacy in gender retrieval within a PWI paradigm. To this end, we conducted two PWI experiments with native speakers of EP (see Sá-Leite et al., 2021). In both experiments, bare nouns were used as a naming instruction. This allowed us: a) to test the tenets of the GAP hypothesis (Sá-Leite et al., 2019) regarding the link between gender transparency and gender effects in the absence of an agreement context; and (b) to analyse how animacy impacts the performance of participants as predicted by its unique nature in terms of semantics (Branigan et al., 2008) and the Animate Monitoring hypothesis (New et al., 2007).

As one of our main purposes was to directly study grammatical gender selection during the production of animate nouns, for the sake of simplicity we focused on the use of animate targets, rather than distractors, which were kept inanimate. Therefore, we manipulated the percentage of animate targets included within each experimental list, emulating previous literature in which different amounts of animate nouns were included as stimuli (see Table 1 for the main conditions within each experiment). In Experiment 1, three different percentages of animate target nouns conveyed three different conditions: 0, 50, and 100%. Since EP is a Romance language with a very high degree of phonological gender transparency (~75% of the Portuguese nouns end in “-o” or “-a,” and 85% of these are transparent [calculated through the Procura-PALavras lexical database, P-PAL, Soares et al., 2018]), it fits the postulates of the GAP hypothesis, by which nouns should retrieve their gender value even in the absence of an agreement context (Sá-Leite et al., 2019). Hence, following the tenets of the GAP hypothesis, as well as the results obtained by Cubelli et al. (2005) and Paolieri et al. (2010a, 2011), we expected to obtain gender competitive effects if the percentage of animate targets was low enough. Yet, the direction of these effects is not clear: a GCE is predicted by the GAP hypothesis, but the Double-Selection model predicts a GIE. As the percentage of animates varied across conditions, we expected the size of gender effects also to vary accordingly, as anticipated by either the semantic prioritization of animate nouns or the Animate Monitoring hypothesis. Both approaches, although for different reasons, predict the same outcome: the higher the quantity of animate targets, the smaller the chance of finding competitive gender effects, or the smaller the effect sizes. In this sense, we expected no gender effects for the 100% animate condition, and indeed this was the case. Yet the condition of 50% did not show gender effects either. An analysis of the whole set of responses across the three conditions with the factor “target animacy” reveals that gender competitive effects were restricted to inanimate targets. In Experiment 2, we conducted another PWI task, this time with 25% of animate targets, to better understand when gender effects are obtainable within a PWI paradigm as a function of the percentage of animate targets within the list. The results failed to reach significance again. We will discuss two possible hypotheses that may be explaining our results and propose future research ideas to put them to the test. The data and scripts used in the study are available at the following link: https://osf.io/8px2z/.

**Table 1:** Conditions in each of the experiments.

	**Animacy presence**	**Target gender**	**Gender congruency**	**Example (target-distractor)**
Experiment 1	0% animate nouns	Masculine	Gender congruent	Casaco-moinho [jacket-windmill]
			Gender incongruent	Casaco-barriga [jacket-belly]
		Feminine	Gender congruent	Mesa-praia [table-beach]
			Gender incongruent	Mesa-palco [table-stage]
	50% animate nouns	Masculine	Gender congruent	Queijo-sismo [cheese, earthquake]
			Gender incongruent	Queijo-relva [cheese-grass]
		Feminine	Gender congruent	Vaca-corda [cow-rope]
			Gender incongruent	Vaca-figo [cow-fig]
	100% animate nouns	Masculine	Gender congruent	Sapo-trigo [toad-wheat]
			Gender incongruent	Sapo-rolha [toad-cork]
		Feminine	Gender congruent	Cabra-túnica [goat-robe]
			Gender incongruent	Cabra-astro [goat-star]
Experiment 2 (25% animate nouns)		Masculine	Gender congruent	Dinossauro-telescópio [dinosaur-telescope]
			Gender incongruent	Dinossauro-ferramenta [dinosaur-tool]
		Feminine	Gender congruent	Bicicleta-camisola [bike-sweater]
			Gender incongruent	Bicicleta-catálogo [bike-catalog]

### Experiment 1

#### Method

##### Participants

Thirty-six native EP students from the University of Minho (31 female; *M*_age_ = 19.53 years, *SD* = 1.65) participated in the experiment and were rewarded with extra course credits. All signed informed consent for experimentation with human subjects previously approved by the Ethics Council of the University of Minho (CEICSH 052/2019). None was moderately or highly proficient in another language with grammatical gender.

##### Materials

Pictures were selected from the International Picture Naming Project (IPNP) database (Szekely et al., 2004). We created a factor of animacy presence featuring three conditions. For the condition of 0% animates, 40 pictures representing inanimate objects were selected. For the condition of 100% animates, another 40 pictures representing animate entities were selected. For the third condition of 50% animates, to maintain the control across conditions and types of pictures, 48 pictures from the previous two conditions (24 inanimates and 24 animates) were selected. Targets were distributed evenly across gender values within each of the three conditions, so that half of them were masculine and the other half feminine. In the condition of 50%, animacy was taken into consideration, and hence half of the animate nouns were masculine (e.g., “*morcego*” [bat]), the other half feminine (e.g., “*raposa*” [fox]) and the same applied to inanimate nouns (e.g., “*sapato*” [shoe] and “*toalha*” [towel]). Phonological gender transparency was controlled so that, in the 0 and 100% conditions, two feminine nouns were opaque (e.g., “*avestruz*” [ostrich]), and the other 18 were transparent for gender (e.g., “*raposa*”), whereas two masculine nouns were opaque (e.g., “*peixe*” [fish]), and the other 18 were transparent for gender (e.g., “*morcego*”). In the 50% condition, we maintained the same proportion of transparent and opaque nouns across the genders of targets (22 transparent and 2 opaque per gender), but from the 4 opaque nouns, one of the opaque feminine nouns and one of the opaque masculine nouns were animate (“*avestruz*” [F] and “*peixe*” [M]), and the other two were inanimate (“*chaminé*” [F, chimney] and “*tapete*” [M, rug]). Nouns with natural (i.e., optional) gender were not used. See the Appendix for a list of the materials (Tables A1,A2).

Pictures within the three conditions of animacy presence were matched for visual complexity (objectively defined by the digital size of the drawing) and goodness-of-depiction (i.e., how well each picture illustrated the target nouns), as obtained from the dataset in Szekely et al. (2005). Target words within each condition were matched across genders on multiple variables, as displayed in Table 2: per-million frequency, number of phonological and orthographic neighbors (N), word length (in letters), and mean logarithmic bigram frequency (these values were taken from the P-PAL database; Soares et al., 2018); per-million frequency (Log10) values were also obtained from the SubtLex-PT database (Soares et al., 2015). Also, values of imageability and concreteness were obtained from the Minho Word Pool database (MWP; Soares et al., 2017). All *ps* > 0.115. Likewise, these variables were controlled: (1) in the 50% condition, taking into consideration the gender of the picture and also the animacy of the picture as an extra factor (*p* > 0.120), and (2) across the three conditions of animacy presence (all *ps* > 0.102). Importantly, differences between the conditions of animacy presence on imageability and concreteness were analyzed with 89% of the targets, as the remaining 11% were not present in the MWP database (see Table 2 for mean values of every controlled variable per target type). The analysis regarding the control of all the target pictures and nouns was conducted through a one-way analysis of variance (ANOVA) considering all the levels of the factors involved (within conditions of animacy presence: “target gender” and “gender congruency,” plus “animacy status of the target” for the condition of 50% animates; and between conditions of animacy presence: “target gender,” “gender congruency,” and “animacy presence”).

**Table 2:** Mean and standard deviations (in parenthesis) of the controlled variables per Target type for all the experimental pictures in Experiment 1.

	**Animacy 0%**		**Animacy 50%**		**Animacy 100%**	
	**Masculine**	**Feminine**	**Masculine**	**Feminine**	**Masculine**	**Feminine**
FpM (log)	3.13 (0.54)	3.10 (0.54)	2.85 (2.35)	2.79 (0.39)	2.84 (0.72)	2.80 (0.63)
PN	4.20 (6.53)	5.15 (5.86)	2.75 (5.58)	4.67 (5.78)	4.05 (6.80)	3.30 (4.16)
ON	4.15 (5.59)	6.60 (7.42)	2.29 (4.16)	3.92 (5.24)	3.20 (4.83)	2.45 (3.40)
L	6.20 (1.70)	6.25 (1.52)	7.04 (1.96)	6.58 (1.52)	6.95 (1.92)	6.30 (1.39)
MLBF	2.57 (0.49)	2.53 (0.49)	2.39 (0.39)	2.36 (0.44)	2.50 (0.31)	2.31 (0.40)
VC	17300.15 (9922.55)	16620.15 (6975.19)	18012.25 (8685.89)	18219.13 (8023.40)	16695.60 (5653.05)	18345.10 (7573.65)
GoD	5.58 (0.76)	5.97 (0.63)	5.97 (0.54)	6.00 (0.64)	5.96 (0.63)	6.07 (0.45)
I	5.71 (1.03)	5.99 (0.40)	6.13 (0.29)	6.01 (0.34)	6.04 (0.36)	6.07 (0.36)
C	6.39 (0.89)	6.58 (0.18)	6.61 (0.20)	6.60 (0.20)	6.52 (0.22)	6.57 (0.18)

*FpM (log), Frequency per million (Log_10_). Obtained from the SubtLex-PT database (Soares et al., 2015). PN, Phonological number of neighbors (N); ON, Orthographic number of neighbors (N); L, Length (in letters); MLBF, Mean log bigram frequency. All obtained from the P-PAL database (Soares et al., 2018). VC, Visual complexity; GoD, Goodness-of-depiction. All obtained from International Picture Naming Project (IPNP) database (Szekely et al., 2004). I, Imageability; C, Concreteness. Obtained from the Minho Word Pool database (Soares et al., 2017). The mean values for imageability and concreteness were calculated using the 82.5, 87.5, and 97.5% of target nouns of condition 0, 50, and 100%, respectively, as the rest of nouns were not available in the database*.

For each picture, four EP distractor nouns were selected (160 for each of the 0 and 100% conditions of animacy presence; 192 for the 50% condition). Two of these had the same grammatical gender as that of the picture (gender congruent condition), and the other two had different grammatical gender (gender incongruent condition). In addition, we controlled the transparency category of the distractors. Hence, from the four distractor nouns selected per target, one of the masculine distractors had the same transparency category as that of the target (e.g., both were transparent) and the other one had a different transparency category (e.g., target was transparent and distractor opaque or vice-versa). The same applied to feminine distractors. All distractors were inanimate nouns. Note that all target-distractor pairs were from different semantic categories, and there was no significant orthographic overlap between them across conditions, according to the NIM database (Guasch et al., 2013; all *ps* > 0.113 within conditions of animacy and *p* > 0.320 between conditions of animacy). Importantly, to avoid effects of facilitation during the naming task, all targets and their respective distractors differed in their initial phoneme (Schiller, 2004; Mousikou et al., 2010; Kinoshita and Verdonschot, 2020).

Targets were assigned to two experimental conditions as a function of their relation in gender congruency (GC) with the distractor. At the same time, a proportional distribution on transparency congruency was maintained for reasons of control. For instance, with the feminine transparent target “*abelha*” (bee), four distractors were selected: (a) congruent in gender and in transparency category, such as “*peruca*” (wig); (b) incongruent in gender but congruent in transparency, such as “*estojo*” (pencil case); (c) congruent in gender but incongruent in transparency, such as “*hélice*” (propeller); and (d) incongruent in both gender and transparency category, such as “*quiosque*” (kiosk). The same was applied to opaque feminine targets and to transparent and opaque masculine ones.

Distractors were matched within the three conditions of animacy presence through a one-way ANOVA conducted with the combination of each of the two levels of “target gender” and “gender congruency” on the number of phonological and orthographic neighbors (N), and word length (in letters), this obtained from the P-PAL database (Soares et al., 2018) and on imageability and concreteness, this obtained from the MWP (Soares et al., 2017) (see mean values within each condition in Table 3, all *ps* > 0.08). Distractors were also matched between the three conditions of animacy presence in the same variables through a one-way ANOVA conducted with the combination of all the levels on the factors “target gender,” “gender congruency between target and distractor,” and “animacy presence” (all *ps* > 0.161). Importantly, the analysis revealed significant differences in the number of orthographic neighbors and in the number of letters (*p* = 0.017 and *p* = 0.018, respectively), yet Bonferroni *post-hoc* pair-wise comparisons actually revealed no significant differences between any conditions (all *ps* > 0.07). Importantly, as discussed in the results, all variables plus logarithmic per million frequency as obtained by the SubtLex-PT database (Soares et al., 2015) were tested as fixed effects in the models of the analyses and none interacted with any of the effects.

**Table 3:** Mean and standard deviations (in parenthesis) of the controlled variables for the distractors across conditions of target gender and gender congruency within every condition of animacy presence in Experiment 1.

	**Animacy 0%**				**Animacy 50%**				**Animacy 100%**			
	**MGC**	**MGI**	**FGC**	**FGI**	**MGC**	**MGI**	**FGC**	**FGI**	**MGC**	**MGI**	**FGC**	**FGI**
FpM (log)	2.80 (0.72)	2.70 (0.68)	2.77(0.58)	2.63 (0.68)	2.56 (0.58)	2.44 (0.70)	2.46 (0.59)	2.34 (0.56)	2.52(0.75)	2.26 (0.84)	2.34 (0.86)	2.37 (0.77)
PN	3.50 (4.30)	3.43 (4.49)	4.68 (4.93)	4.30 (4.24)	2.21 (2.91)	2.31 (3.48)	3.73 (4.11)	3.35 (3.79)	3.15 (4.43)	3.55 (5.00)	2.65 (2.59)	2.68 (3.54)
ON	3.13 (4.05)	3.40 (4.11)	4.53 (4.87)	4.43 (4.81)	1.75 (2.40)	2.04 (2.97)	3.52 (4.30)	3.02 (3.92)	2.53 (3.42)	3.05 (4.24)	2.40 (3.04)	2.20 (3.04)
L	6.20 (1.71)	6.15 (1.64)	6.13 (1.64)	5.95 (1.52)	7.08 (1.71)	6.92 (1.83)	6.56 (1.39)	6.56 (1.39)	6.93 (1.77)	6.90 (11.89)	6.53 (1.33)	6.40 (1.33)
I	5.62 (0.70)	5.40 (1.20)	5.40 (1.08)	5.47 (0.89)	5.68 (0.82)	5.34 (1.01)	5.63 (0.82)	5.56 (0.82)	5.69 (1.08)	5.51 (1.20)	5.36 (1.45)	5.59 (0.89)
C	5.89 (1.08)	5.56 (1.77)	5.52 (1.58)	5.92 (0.89)	6.03 (1.08)	5.68 (1.64)	5.96 (1.20)	5.91 (1.14)	6.00 (1.14)	5.85 (1.71)	5.73 (1.90)	5.88 (1.20)
OV	0.15 (0.06)	0.12 (0.13)	0.14 (0.06)	0.15 (0.13)	0.16 (0.06)	0.13 (0.06)	0.14 (0.06)	0.13 (0.06)	0.14 (0.06)	0.14 (0.13)	0.14 (0.13)	0.09 (0.06)

*M and F, Masculine and Feminine; GC and GI, Gender Congruent and Gender Incongruent; FpM (log), Frequency per million (Log_10_). Obtained from the SubtLex-PT database (Soares et al., 2015). PN, Phonological number of neighbors (N); ON, Orthographic number of neighbors (N); L, Length (in letters). All obtained from the P-PAL database (Soares et al., 2018). I, Imageability; C, Concreteness. Obtained from the Minho Word Pool database (Soares et al., 2017). OV, Orthographic overlap between targets and distractors. Obtained from the NIM database (Guasch et al., 2013). The mean values for imageability and concreteness were calculated using the 66, 54, and 51% of distractor nouns of condition 0, 50, and 100%, respectively, as the rest of nouns were not available in the database. Note that the values for FpM (log) showed significant differences in the ad hoc ANOVA but the variable was included in the final models and did not hold any type of impact on the results*.

Finally, as training items, eight different pictures were selected from the IPNP database, along with eight new distractors. Within each condition of animacy presence, four different lists were created for counterbalancing purposes. Each of these featured the same conditions based on the target gender (masculine/feminine) and the gender congruency between target and distractor (congruent/incongruent), including the 40 (0 and 100% of animacy presence) or 48 target pictures (50% of animacy presence). Each target picture was presented four times within each condition of animacy presence, one time per list and each time associated with a different distractor. Participants were randomly assigned to one of the four lists, assuring the same number of participants per list.

##### Procedure

Participants engaged individually in the three different conditions of animacy presence (0, 50, and 100%, of animate targets), i.e., all did the three conditions, these separated by a month and a half time lapse. We guaranteed that the first condition for all participants was the one containing no animate target, since we wanted to avoid any kind of confounding effects of animacy emerging from previous exposure to the task with pictures showing animate entities. Given that the condition of 50% animate targets contained the stimuli from the other two conditions, we also considered that this should be the last one to be conducted, so that animate and inanimate targets were not disproportional in their novelty for the participants. After this, a familiarization phase and an experimental task took place in a soundproof booth, and these were subsequently repeated for each condition, but with different materials. Participants signed a consent form for the whole experiment before the first session.

The familiarization phase consisted of the presentation of all the pictures involved in the subsequent experimental task to guarantee high naming agreement scores. Each picture was presented automatically for 2 s in a single slide, along with the nouns that participants would have to use to name them in the task. Words were shown in lowercase letters (Agency FB 36) below the respective picture. The presentation was done in Microsoft Corporation (2010). No overt response was required. Stimulus presentation was randomized across participants.

The experimental task consisted of a PWI paradigm, in which participants were instructed to name each picture using a bare noun (“*mesa*”) as quickly and accurately as possible, while ignoring the distractor noun superimposed onto the picture. All the pictures were presented at the center of the computer screen. The distractors, presented at the center of the pictures with a 0 ms SOA, were shown in lowercase letters in Courier New font, 14 point (see Cubelli et al., 2005 for a similar procedure). Experimental trials followed this structure: (a) a fixation point (+) at the center of the computer screen, for 500 ms; (b) the target picture with the superimposed distractor for 2,000 ms or until response; (c) a blank space for 500 ms as an inter-trial interval. Participants first performed a training block of 8 trials. Experimental trials were presented pseudo-randomly per participant following the next rules: only 2 targets of the same gender congruency condition, the same target gender condition, and the same controlled transparency congruency variable (transparent/congruent targets and distractors) could appear consecutively. RTs were measured from the onset of the stimulus to the beginning of the naming response. Stimulus presentation was done using the DMDX software (Forster and Forster, 2003). Naming RTs were recorded by the voice-key from the presentation of the target to the onset of the naming response and then checked offline using the CheckVocal software (Protopapas, 2007).

The session for each condition of animacy lasted ~10 min.

#### Results and Interim Discussion

We discarded 491 data points (10.66% of the total) from the analyses: those containing incorrect responses (357 data points, 7.75% of the total), and those with RTs that exceeded 2.5 SD of each participant's mean (134 data points, 2.91% of the total). Data were analyzed by means of linear mixed-effect models (e.g., Baayen, 2008; Baayen et al., 2008). We used the lme4 package from R (Bates et al., 2014). Different linear models were created to examine the effect of the variables of interest (animacy presence, target gender and gender congruency) and their interactions on inverse RTs (−1000/RT). These variables and their interactions were introduced as fixed effects. In addition, each model included random intercepts and random slopes by participants and items, following a maximal random effects structure (Barr et al., 2013). In most cases, we were unable to get the maximal random effects structure to converge, so we kept only those random slopes that allowed for convergence. In particular, the random structure was stepwise simplified by removing in each step the effect that explained the smallest proportion of the variance, starting from the most complex model (i.e., that including the three-way interaction in Experiment 1), until a convergent model with at least one critical effect on the random slopes was obtained. To determine the significance of fixed effects, log-likelihood ratio tests were used (R function Anova). We assessed the contribution of each fixed effect by comparing a model that included the effect of interest with another model that did not include it. *P*-values for pairwise comparisons were estimated using the lmerTest package (Kuznetsova et al., 2014)^4^.

Table 4 shows the mean RTs for each condition. The interaction between congruency and animacy reached significance, χ^2^(2) = 7.51, *p* = 0.023. To examine this interaction, we analyzed the effect of gender congruency in each of the conditions of animacy presence. There was a GCE in the 0% animates condition, *Estimate* = −0.029, *SE* = 0.011, *p* = 0.008, χ^2^(2) = 7.15, *p* = 0.007, but no effects of congruency were observed in the other two animacy conditions (all *p*s > 0.292). In addition, we found a significant effect of animacy, χ^2^(2) = 26.45, *p* < 0.001, indicating that RTs for the 50% animates condition were faster than both RTs for 0% animates condition, *Estimate* = −0.082, *SE* = 0.016, *p* < 0.001, and RTs for the 100% animates condition, *Estimate* = −0.087, *SE* = 0.018, *p* < 0.001, probably due to an effect of familiarization with the viewed images (note that the 50% condition was composed of stimuli taken from the other two conditions and was the last carried out by the participants). No other main effects or interactions were observed.

**Table 4:** Mean reaction times and standard errors (SE) for each condition in Experiment 1.

**Animacy**	**Gender**	**Congruency**	**Mean**	***SE***	***GCE size***
0%	Masculine	Incongruent	879	12.00	
		Congruent	856	11.10	
	Feminine	Incongruent	880	11.50	
		Congruent	855	11.60	
					24 ms
50%	Masculine	Incongruent	829	10.40	
		Congruent	815	8.87	
	Feminine	Incongruent	837	9.96	
		Congruent	851	10.80	
					0 ms
100%	Masculine	Incongruent	896	11.60	
		Congruent	909	12.30	
	Feminine	Incongruent	918	12.50	
		Congruent	917	12.00	
					−6 ms

Animacy, factor “Animacy presence,” Gender, factor “Target gender,” Congruency, factor “Gender congruency.” Formula of the final model: −1000/RT ~ congruency*animacy*gender + (animacy | participant) + (congruency | item).

A possible explanation for the congruency effect found in the 0% animate condition, but not in the 50 and 100% animate ones, might be that animate and inanimate targets were not perfectly matched in their lexical and semantic properties. We tested this hypothesis by examining the interaction between “gender congruency” and “target animacy” (animate vs. inanimate targets rather than the percentage of animates in the stimuli list) on the whole set of responses from the three conditions after controlling for all these variables. We created a linear mixed-effects model in which we introduced all lexical (e.g., frequency, number of neighbors, etc.) and semantic (e.g., concreteness) variables of the target words and pictures (e.g., visual complexity, goodness-of-depiction), together with the interaction between gender congruency and target animacy, as predictors of RTs. We also introduced participants and items as random effects, and gender congruency in the item random slope (any more complex structure of random effects did not allow for model convergence). The results showed an interaction between congruency and animacy, χ^2^(1) = 4.92, *p* = 0.027. A significant congruency effect was observed for inanimate targets, *Estimate* = −0.09, *SE* = 0.03, *p* = 0.003, χ^2^(1) = 5.10, *p* = 0.024, but this effect was not significant for animate targets, χ^2^(1) = 0.82, *p* = 0.366. This has two strong implications: (1) It confirms that indeed, animate and inanimate targets may behave differently and that inanimate targets show gender competitive effects whereas animate ones do not; (2) it suggests that the results of this experiment do not seem to be due to the effect of the variables in which the animate and inanimate targets (and distractors) vary.

The GCE found in the 0% animates list and the GCE found for inanimate targets considering the three conditions support the tenets of the GAP hypothesis according to which gender competitive effects should be found in a transparent language such as PE without the presence of an agreement context. It also supports the predicted direction of the effect (congruency) and goes against the GIE predicted by the Double Selection model. Hence, the results are favorable to the idea that gender is represented through two gender nodes that accumulate activation and compete for selection in a grammatical stratum of lexical access. On the other hand, as predicted by the semantic prioritization hypothesis by which animacy could be responsible for skipping gender selection and by the Animate Monitoring hypothesis by which the degree of attention given to animate pictures could diminish the competitive role of the distractors, there were no competition between gender values of target and distractors for animate targets. Surprisingly, there was not even an effect for the list with 50% of animate and inanimate targets. In order to test which is the minimum allowable percentage of animates within the stimuli list for gender effects to be obtained, we conducted a second experiment featuring 25% of animate targets, to comprise a greater range of cases and understand to what extent prior studies could have been affected by the random inclusion of animate targets.

### Experiment 2

#### Method

##### Participants

Forty-eight native EP students from the same population as that of Experiment 1 (46 female; *M*_age_ = 21.32 years, *SD* = 3.88) participated in this experiment and were rewarded with extra course credits. All signed informed consent for experimentation with human subjects previously approved by the Ethics Council of the University of Minho (CEICSH 052/2019). None was moderately or highly proficient in another language with grammatical gender.

##### Materials

We selected 48 pictures from Experiment 1: twenty-five percent of them (12) were animate and the other 75 percent (36) were inanimate. We selected 4 distractors per picture (a total of 192 distractors), following the same assignment rules previously described and keeping the proportion between transparent and opaque nouns (for the whole list of materials, see the Appendix, Table A3). Pictures were controlled across gender values (masculine and feminine) for complexity and goodness of depiction (*p*s > 0.126); when considering the factor Animacy (animates and inanimates) along with Target Gender, pictures also showed no significant differences in those variables (*p*s > 0.434). As in Experiment 1, target nouns were matched across gender values on the variables: per-million frequency (Log10), this obtained from the SubtLex-PT database (Soares et al., 2015); number of phonological and orthographic neighbors (N), word length (in letters), and mean logarithmic bigram frequency, these obtained from the P-PAL database (Soares et al., 2018) and finally imageability and concreteness, these obtained from the Minho Word Pool database (MWP; Soares et al., 2017). All *p*s > 0.096 (see Table 5 for mean values). Note that the imageability and concreteness variables were only controlled for 85% of the target nouns, for which values were found in the Minho Word Pool database. Of interest, targets were also controlled when considered not only across gender values but also across animates and inanimates (all *p*s > 0.267). All these analyses were based on one-way ANOVAS.

**Table 5:** Mean and standard deviations (in parenthesis) of the controlled variables across targets' gender values for all the experimental pictures in Experiment 2.

	**Masculine**	**Feminine**
FpM (log)	3.02 (0.49)	2.95 (0.49)
PN	3.42 (5.98)	5.46 (6.07)
ON	3.42 (5.29)	5.67 (7.15)
L	6.58 (2.01)	6.38 (1.57)
MLBF	2.45 (0.44)	2.45 (0.49)
VC	16067.79 (5355.32)	18857.88 (7939.83)
GoD	5.95 (0.59)	6.08 (0.44)
I	5.88 (0.93)	6.00 (0.34)
C	6.44 (0.78)	6.58 (0.18)

*FpM (log), Frequency per million (Log_10_). Obtained from the SubtLex-PT database (Soares et al., 2015). PN, Phonological number of neighbors (N); ON, Orthographic number of neighbors (N); L, Length (in letters); MLBF, Mean log bigram frequency. All obtained from the P-PAL database (Soares et al., 2018). VC, Visual complexity; GoD, Goodness-of-depiction. All obtained from International Picture Naming Project (IPNP) database (Szekely et al., 2004). I, Imageability; C, Concreteness. Obtained from the Minho Word Pool database (Soares et al., 2017). The mean values for imageability and concreteness were calculated using 85% of the target nouns, as the rest of nouns were not available in the database*.

Distractors were controlled across conditions of gender congruency and the target's gender value for the variables number of phonological and orthographic neighbors (N), and word length (in letters), these obtained from the P-PAL database (Soares et al., 2018), and on imageability and concreteness (115, i.e., 60% of the distractors), these obtained from the MWP (Soares et al., 2017) (see mean values within each condition in Table 6, all *p*s > 0.152). Besides, all target-distractor pairs differed in their initial phoneme, were from different semantic categories, and there was no significant orthographic overlap between them across conditions, according to the NIM database (Guasch et al., 2013; *p* > 0.710). All these analyses were based on one-way ANOVAS. Importantly, as said in the previous experiment, all variables plus logarithmic per million frequency as obtained by the SubtLex-PT database (Soares et al., 2015) were tested as fixed effects in the models of the analyses and none interacted with any of the effects.

**Table 6:** Mean and standard deviations (in parenthesis) of the controlled variables for the distractors across conditions of target gender and gender congruency in Experiment 2.

	**MGC**	**MGI**	**FGC**	**FGI**
FpM (log)	2.76 (0.46)	2.54 (0.69)	2.64 (0.55)	2.51 (0.61)
PN	3.04 (4.02)	3.08 (4.16)	4.44 (4.71)	4.35 (4.43)
ON	2.63 (3.60)	3.04 (3.46)	4.10 (5.06)	4.02 (4.71)
L	6.48 (1.73)	6.56 (1.94)	6.42 (1.59)	6.10 (1.52)
I	5.65 (0.83)	5.28 (1.18)	5.52 (0.97)	5.45 (0.90)
C	6.01 (1.04)	5.43 (1.80)	5.77 (1.45)	5.97 (0.97)
OV	0.14 (0.07)	0.17 (0.35)	0.13 (0.07)	0.12 (0.07)

*M and F, Masculine and Feminine. GC and GI, Gender Congruent and Gender Incongruent. FpM (log), Frequency per million (Log_10_). Obtained from the SubtLex-PT database (Soares et al., 2015). PN, Phonological number of neighbors (N); ON, Orthographic number of neighbors (N); L, Length (in letters). All obtained from the P-PAL database (Soares et al., 2018). I, Imageability; C, Concreteness. Obtained from the Minho Word Pool database (Soares et al., 2017). OV, Orthographic overlap between targets and distractors. Obtained from the NIM database (Guasch et al., 2013). The mean values for imageability and concreteness were calculated using 60% of the distractor nouns, as the rest of nouns were not available in the database. Note that the values for FpM (log) showed significant differences in the ad hoc ANOVA but the variable was included in the final models and did not hold any type of impact on the results*.

The same 8 training stimuli featured in Experiment 1 were also selected here. Four different lists were created for counterbalancing purposes. Each of these featured the same conditions based on the target gender (masculine/feminine) and the gender congruency between target and distractor (congruent/incongruent). Each target picture was presented four times, one time per list and each time associated with a different distractor. Participants were randomly assigned to one of the four lists, assuring the same number of participants per list.

##### Procedure

We followed the same procedure as that of Experiment 1, except that it was only one PWI task as there was no factor of animacy presence.

#### Results

We excluded 254 data points (11.03% of the total) from the analyses: those corresponding to incorrect responses (178 data points, 7.73% of the total), and those containing RTs that exceeded 2.5 SD of each participant's mean (76 data points, 3.30% of the total). The data analyses were identical to those performed in Experiment 1.

The mean RTs for each condition are presented in Table 7. No main effect or interaction reached significance. This came out as surprising: with only 25% of animate targets within the stimuli list, the GCE disappeared, even though gender competitive effects have been observed with similar percentages of animate targets (e.g., Cubelli et al., 2005; Paolieri et al., 2010a, 2011). The fact that such a small percentage of animate targets may be behind this absence gives animacy a greater impact than we could have imagined when inspecting both the theoretical and experimental literature. To be sure of the null results, we examined the data through Bayesian analyses. We used the Bayes Factor (BF_10_), which allowed us to quantify the amount of evidence for (H_1_) and against (H_0_) the effect of the variables of interest. The magnitude of this evidence is presented as an odds ratio (H_1_ evidence/H_0_ evidence), which can range from 0 to infinite. If the value increases, it provides evidence in favor of H_1_; if it approaches 0, it provides evidence in favor of H_0_. Values close to or equal to 1 indicate that both H_1_ and H_0_ are equally probable. By convention, values above 3 can be interpreted as moderate evidence supporting the H_1_, and values below 1/3 give moderate support for the H_0_ (Jeffreys, 1961; Dienes, 2014). We used the BayesFactor package of R (Morey and Rouder, 2015) to perform these analyses. We compared a model that included the factor of interest (H_1_) with one that did not (H_0_) using the lmBF function. Here we will use BF01 to indicate the amount of evidence in favor of H0 relative to H1 (in which the interpretation is inverted, i.e., BF01 of 3 would suggest moderate evidence for H0 and 1/3 moderate evidence for H1, and so on.). A JZS prior with scaling factor r = 0.707 was used in all analyses (Rouder et al., 2009). The effect of gender congruency, χ^2^(1) = 0.04, *p* =0.832, BF_01_ = 10.86, gender, χ^2^(1) = 0.64, *p* = 0.425, BF_01_ = 4.04, and the interaction between gender congruency and target gender, χ^2^(1) = 3.04, *p* = 0.081, BF_01_ = 4.15, were not significant and the Bayesian analyses suggest that the evidence for the null hypothesis is large enough, supporting the absence of effects in this experiment. Although a direct comparison between animate and inanimate targets as the one conducted with all the lists in Experiment 1 was not possible due to the low amount of animate targets, we additionally examined the effect of gender congruency exclusively for inanimate targets. As in the analysis with all targets, the effect of gender congruency was not significant, χ^2^(1) = 0.22, *p* = 0.637, BF_01_= 15.66. Overall, the results contrast with those from other studies testing Romance languages in which a GIE was obtained (Cubelli et al., 2005; Paolieri et al., 2010a, 2011) and the percentage of animate stimuli, and particularly targets, was similar to the percentage featured in this experiment (10 to 33.33% of animate targets plus animate distractors with unknown repercussions for gender processing). Two different but perhaps complementary underlying reasons for our results can be advanced here. On the one hand, the GCE has been shown to be a quite small and slippery effect both in bilingual and monolingual populations (bilingual GCE: *g* = 0.24, *SE* = 0.04, *p* < 0.001; monolingual GCE: *g* = 0.102, *SE* = 0.042, *p* = 0.019; Sá-Leite et al., 2020, under review). The absence of an effect with the inanimate targets of our additional analysis may be an indication of the lack of robustness of the effect. In fact, following the GAP hypothesis, gender effects should be guaranteed in EP when an agreement context is present. Yet, relying on the degree of activation reached by nouns themselves to observe a small effect like the GCE may be tricky. The GCE seems to be highly sensitive to the list composition, and these 25% of animate targets could have indeed diminished our chance to observe any GCE in this experiment. Hence, a second possible cause behind the present results may be a carry-over effect induced by animate targets. We will consider this in more detail in the Discussion.

**Table 7:** Mean reaction times and standard errors (SE) for each condition in Experiment 2.

**Gender**	**Congruency**	**Mean**	***SE***	***GCE size***
Masculine	Incongruent	848	8.76	
	Congruent	862	9.41	
Feminine	Incongruent	847	8.81	
	Congruent	836	8.66	
				−2 ms

Gender, factor “Target gender,” Congruency, factor “Gender congruency.” Formula of the final model: −1000/RT ~congruency*gender + animacy + (congruency | participant) + (congruency | item).

## General Discussion

In the present study, we aimed to examine the gender (in)congruency effect in interaction with the animacy of the stimuli in an attempt to better understand the representation and processing of grammatical gender during language production. To do that two PWI experiments with EP bare nouns were conducted. On the one hand, these experiments would allow us to test the tenets of the GAP hypothesis (Sá-Leite et al., 2019) according to which the production of bare nouns in a gender transparent language (Velnić, 2020) should entail the selection of gender. On the other hand, they would also allow us to test the impact of animacy on the predicted gender competitive effects, following the tenets of the semantic prioritization that occurs in the lexical access of animate nouns (Branigan et al., 2008) and those of the Animate Monitoring hypothesis (New et al., 2007). We expected to obtain competitive gender effects in the form of a GCE as predicted by the GAP hypothesis (Sá-Leite et al., 2019). However, taking into account previous studies with transparent Romance languages (Italian: Cubelli et al., 2005; Italian and Spanish: Paolieri et al., 2010a, 2011), these gender competitive effects could emerge in the form of a GIE as predicted by the Double-Selection model (Cubelli et al., 2005). In any case, gender competitive effects would be observed only if the percentage of animate targets was low enough, as animacy may induce the skipping of gender selection or/and capture the attention of the participants, thus diminishing activation and the competitive role of the distractors.

In the first experiment, different percentages of animate target nouns were tested, with all distractors inanimate. The condition of 0% animate targets was critical, as it provided the ideal conditions to observe competitive gender effects. Indeed, competitive gender effects were obtained, although in the form of congruency rather than incongruency. This supports the main premise of the GAP hypothesis and its proposed structure of lexical access regarding the organization of grammatical gender as gender nodes that accumulate activation and compete for selection. These competitive effects also suggest that the target noun is not only activating a gender node during lexical access but also selecting it, which triggers competition for selection with the gender node activated by the distractor. Crucially, this happened without the presence of an agreement context, contrarily to what is observed with Germanic and Slavic languages (e.g., Schriefers, 1993; Schiller and Caramazza, 2003). The results, hence, constitute supporting evidence for the idea that transparent languages activate and select gender at the level of grammatical encoding during bare noun production. However, they go against the GIE predicted by the Double-Selection model (Cubelli et al., 2005), which was previously obtained in transparent languages that are similar to EP, such as Spanish and Italian (Cubelli et al., 2005; Paolieri et al., 2010a, 2011). The Double-Selection model describes grammatical gender as an inherent characteristic of nouns located at a lemma level that integrates both conceptual and grammatical features. Every word would have its own lemma encompassing all its semantic and grammatical features; hence, word lemmas would include their specific gender value as an associated feature and, since they compete for selection by similarity, lemmas of the same gender interfere with each other during lexical access. Our results, though, suggest that gender values are represented by nodes (the masculine and feminine gender nodes) located at a lemma level separated from the level of conceptual encoding as conceived of by the GAP hypothesis. These nodes seem to gather activation until reaching the threshold for selection. The activation of different gender values, and thus different gender nodes, would translate into interference for gender selection. Similarly, nouns of the same gender would contribute to the activation of the same gender value, facilitating its selection. Our results also coincide with those obtained with bilingual populations. Bilinguals have been shown to have an integrated gender system with both languages sharing the same gender nodes (Sá-Leite et al., 2019, 2020). In many studies, the production of nouns of one gender in the second language is facilitated when their translations in the first language activate the same gender node but hampered when they activate a different gender node (cross-linguistic GCE; Paolieri et al., 2010b, 2019; Morales et al., 2011; Manolescu and Jarema, 2015; Klassen, 2016)—competition does not occur by similarity.

Regarding animacy, results were quite clear: gender competitive effects were restricted to inanimate targets and to the 0% animacy condition. The absence of effects in both the 50% animate condition and in Experiment 2 with 25% of animate targets comes out as striking. Indeed, the GCE has been shown to be a small and heterogeneous effect both in monolingual and bilingual populations (bilingual GCE: *g* = 0.24, *SE* = 0.04, *p* < 0.001; monolingual GCE: *g* = 0.102, *SE* = 0.042, *p* = 0.019; Sá-Leite et al., 2020, under review). In fact, the GAP hypothesis points that when agreement is present, the degree of activation reached by gender nodes should always be enough to entail gender effects. However, when only bare nouns are produced, it states that gender competitive effects *can* be observed, but the degree of activation may not always be enough to entail solid effects. Given that the GCE is a small and heterogeneous effect and that in the absence of an agreement context, the degree of activation may not always be enough to behaviourally reflect competition, it seems then that the GCE can be highly sensitive to the composition of the stimuli list. The analysis of the inanimate targets in Experiment 2 suggests that indeed, the GCE is not robust and that the presence of animate stimuli could hence have decreased our chance to observe it.

We advance two possible reasons underlying the absence of gender effects with animate targets that might also explain why effects disappeared with the inclusion of animate targets in the stimuli list. On the one hand, following the Animate Monitoring hypothesis and its supporting evidence (e.g., New et al., 2007; Altman et al., 2016), animates (especially if represented by pictures) entail an attentional bias and are highly independent of the context. Therefore, the context remains easily ignored by our visual perceptive mechanisms if an animate entity is included. This could result in an attentional deficit for the distractors, causing an absence of effects with animate targets. On the other hand, the explanation could be on the character of animacy from a semantic perspective. More specifically, due to the high amount of resources that are consumed in the first phase of conceptual encoding and the “*special*” status that is given to animates for ontogenetic reasons, if agreement is not necessary, gender selection may be skipped at the lemma level to avoid unnecessary costs as well as avoiding slowing down the response. The idea of skipping the processing of grammatical characteristics when not necessary was already proposed in the IN model (Caramazza, 1997), which states that a response can be given through direct connections from the conceptual to the morpho-phonological stratum. These two hypotheses could explain the absence of gender competitive effects with animate nouns but could also be behind the disappearance of the GCE when animate targets are included in the stimuli list, as they might induce some kind of modulation in the processing of the inanimate stimuli. Following the Animate Monitoring hypothesis (New et al., 2007), animate pictures may be capturing the participants' attention in an accentuated way, so the degree of attention given to the whole set of pictures, including inanimate ones, can also be higher, diminishing the interfering role of the distractors. The same could be said about the semantic prioritization principle of the processing of animate nouns. It may be the case that when gender processing is skipped for some stimuli, it induces the skipping of this characteristic for other stimuli within the same task. Although alterations on the processing of inanimate stimuli seem to better fit the hypothesis based on attentional mechanisms, it is worth mentioning that, when looking at the means for the animacy presence conditions (see Table 4), our RTs suggest that animates indeed require more cognitive resources and hence entail deeper processing, which would suggest prioritization given the rich semantic encoding. Specifically, the mean RTs appear to be higher for the condition of 100% than for the other conditions. Interestingly, in Experiment 1 the condition of 50% animates shows the lowest RTs and these are significantly different from the mean RTs in the conditions featuring 0 and 100% of animates. At first glance, we would expect the condition of 50% to show higher RTs than the condition of 0%, but lower than the condition of 100%. As mentioned above, this result can be explained simply as the effect of practice. The condition of 50% animates was conducted after the conditions of 0 and 100% animates and consisted of half of the stimuli of the other two conditions. Even though each session took place 1 month and a half after the previous one, we believe that this is the most reasonable explanation in both theoretical and experimental terms. If we are right, both animate and inanimate stimuli should have benefited from the earlier presentation of the stimuli in the 100 and 0% animate condition, respectively. We compared the mean RTs of the inanimate stimuli from the 50 and 0% animate conditions, as well as those of animate stimuli from the 50 and 100% animate conditions. The results showed significant differences for both comparisons, in line with the explanation based on an effect of practice (819 vs. 868 ms, 848 vs. 910 ms, respectively, all *ps* < 0.001, see Table 8).

**Table 8:** Mean reaction times and standard errors for animate and inanimate nouns in Experiment 1.

**Animacy Presence**	**Animacy status**	**Mean**	***SE***
50% animates	Animates	848	7.54
	Inanimates	819	6.63
100% animates	Animates	910	6.04
0% animates	Inanimates	868	5.78

*Animacy status is related to the animacy status of the target nouns of each condition of Animacy Presence*.

Importantly, the two hypotheses that we have presented as possible explanations to the absence of gender competitive effects in animate target nouns are not necessarily antagonistic. In this sense, an attentional bias for animates might exist, precluding the observation of any competitive effect; nevertheless, animates may also skip gender processing due to semantic prioritization. Future studies should use other paradigms and experimental techniques (e.g., eye-tracking, event-related potentials) toward disentangling the two alternatives. More specifically, we encourage future studies to examine other type of effects, especially an ortho-phonological facilitation effect with animate targets and inanimate distractors. For instance, in an experiment in Portuguese, one could select animate targets such as “*mac**aco*” (monkey) and pair them with form-related distractors such as “*cas**aco*” (jacket) and unrelated distractors such as “*panela*” (pan). If attentional-driven reasons are behind the absence of results with animate targets in such a study, an ortho-phonological based effect should not be obtained or should at least decrease its size as the quantity of animate targets increases. On the contrary, if semantic-driven reasons are in the roots of our results and the processing of grammar features such as gender is skipped, form effects should emerge regardless of the animacy status of the targets. Additionally, the role of animacy in the GCE and in the PWI itself could also be explored in other languages, to understand to what extent animacy is relevant for gender processing in languages in which agreement contexts have been shown to be mandatory for gender competitive effects to appear. In these cases, it would be interesting assessing the impact of animacy on the size of the GCE.

Finally, it seems unclear to us why the competitive gender effects obtained by Cubelli et al. (2005) and Paolieri et al. (2010a, 2011) are of incongruency rather than congruency. An inspection of their materials allowed us to verify that the percentage of animate nouns in the targets list ranges between 10 and 33.33% and that of the distractors between 5 and 25%, these having been randomly included. This leads us to question the extent to which these results may be an entirely reliable reflection of gender processing, especially if only inanimate nouns are responsible for the effect. It might be worth mentioning that there may be some differences in terms of stimuli control between theirs and our study. In this respect, we would like to highlight the fact that our study had a stricter control across conditions featuring 21 variables related to both targets and distractors^5^. Our experiments were also controlled for gender transparency of targets and distractors, and gender transparency congruency, semantic relatedness, and first phoneme overlap between targets and distractors. On the one hand, this makes us wonder which variable other than animacy itself could be behind our results. On the other hand, if the GCE is indeed a small and heterogeneous effect that is highly sensitive to the composition list, Cubelli et al. (2005) and Paolieri et al. (2010a, 2011) studies could have indeed lacked of the necessary control to obtain reliable results (only 4 to 5 variables were controlled *ad-hoc* [word frequency, length, gender transparency, phonological overlap, and sometimes semantic relatedness], with no testing for inclusion in the models, and with no control between animate and inanimate stimuli). We believe that future research in Italian and Spanish should increase the control of the materials across conditions and feature only inanimate pictures and distractors in the experimental list. Likewise, the specific role of animate distractors in the competitive gender effects should be carefully assessed. In any case, it is worth noting that the “tendency” observed in our 50% condition of Experiment 1 and in Experiment 2, although not significant, was of incongruency (see Tables 4, 7). A closer inspection of data from the two experiments revealed considerable interindividual variability, sometimes depending on the gender of the target, and this may merit attention in future studies. This variability might explain why a GIE is sometimes observed, as in Cubelli et al.'s (2005) and Paolieri et al.'s (2010a, 2011) experiments.

In sum, the present study provides supporting evidence for the idea that transparent languages activate gender nodes during the lexical access of nouns in the absence of an agreement context, as argued by the GAP hypothesis. Importantly, it is the first study to obtain evidence of the impact of animacy on the assessment of competitive gender effects within the PWI paradigm. Animate target nouns showed null effects of gender and their presence within mixed stimuli lists blurred competitive gender effects between targets and distractors. We give credit to two possible explanations behind the results that should be tested in the future, the semantic prioritization of animates and the attentional bias as defined by the Animate Monitoring hypothesis. Further research is now needed to clarify the robustness of the effect and the underlying mechanisms of grammatical gender processing using more sensitive techniques such as electroencephalographic measures in which the time-course of lexical access can be better assessed.

## Data Availability Statement

The original contributions presented in the study are publicly available. This data can be found here: https://osf.io/8px2z/.

## Ethics Statement

The studies involving human participants were reviewed and approved by Ethics Council of the University of Minho (CEICSH 052/2019). The patients/participants provided their written informed consent to participate in this study.

## Author Contributions

AS-L and MC conceived the idea and experimental design of the study. AS-L managed the data collection, was responsible for writing and editing the manuscript overall, and contributed to the main theoretical hypotheses and interpretations. JH analyzed the data and wrote the results sections of the paper. MC and IF reviewed the different drafts of the manuscript. IF was responsible for the funding of the experiment and its publication. All authors made theoretical contributions for the general discussion and approved the final version of the manuscript for submission.

## Conflict of Interest

The authors declare that the research was conducted in the absence of any commercial or financial relationships that could be construed as a potential conflict of interest.

## Footnotes

^1^As an exception, there is a group of animate nouns with natural gender whose form does not vary across values. In this case, the biological sex of the referent is always specified through agreement with the other elements of the speech. For instance, in EP, we must say “the dentist” as either “*o* (M) *dentista* (hence, M)” or “*a* (F) *dentista* (hence, F)”.

^2^But see Vigliocco and Franck (1999) and Vigliocco and Franck (2001) for evidence of no differences between animate and inanimate nouns in the percentage of agreement errors during language production.

^3^Note that in Paolieri et al. (2010a), Paolieri et al. (2011) and Cubelli et al. (2005) studies, experiments with 25% or more of animate targets (*n* = 6 experiments) have an average effect of 15.67 ms whereas those with less than 25% of animate targets (*n* = 7 experiments) show an effect of 30.67 ms.

^4^In each of the three conditions, as well as in the other experiment, we also created a series of models in which we introduced as fixed effects the variables that were controlled between the different conditions (e.g., log word frequency, number of neighbors, etc.; see Materials). In many cases, these models did not converge, and in those cases in which they did, the results did not differ from the models that did not include the controlled variables. In view of this, and for the sake of clarity and simplicity, we decided to present the results of the models that did not include the controlled variables.

^5^These variables included per million logarithmic frequency from the P-PAL database and the Portuguese Sublex, zipf's frequency (P-PAL), number of phonological and number of orthographical neighbors (P-PAL), word length, mean logarithmic bigram frequency (P-PAL), imageability, concreteness and subjective frequency (all UMinho Word Pool). Each of these variables joined the models for both targets, and also for distractors. Visual complexity and goodness-of-depiction joined as variables of the pictures. The orthographic overlap between targets and distractors also joined and was calculated through the NIM database (see Materials).
