# Supplementary material for: Of Beavers and Tables: The Role of Animacy in the Processing of Grammatical Gender Within a Picture-Word Interference Task
Source: Front Psychol. 2021 Jul 8;12:661175. doi: 10.3389/fpsyg.2021.661175 (PMC8295689; doi:10.3389/fpsyg.2021.661175)
Supplement: Supplementary file 1 [file Data_Sheet_1.docx]

**Appendix**

Materials for all the experiments

Table A1

*Target and distractor nouns for Experiment 1 (first and second conditions of animacy presence)*

| **Exp. 1 - First Condition** | | | | | | **Exp. 1 – Second Condition** | | | | | |
| --- | --- | --- | --- | --- | --- | --- | --- | --- | --- | --- | --- |
| **Feminine Targets** | **Distractors** | | **Masculine Targets** | **Distractors** | | **Feminine Targets** | **Distractors** | | **Masculine Targets** | **Distractors** | |
| mesa  (table) | praia  arte  palco  sol | beach  art  stage  sun | carro  (car) | vento  vale  data  fome | wind  valley  date  hunger | mulher  (woman) | análise  cultura  futebol  diálogo | analysis  culture  soccer  dialogue | homem  (man) | filme  livro  fonte  água | movie  book  fountain  water |
| janela  (window) | letra  fraude  navio  exame | letter  fraud  ship  exam | vinho  (wine) | braço  parque  praça  ponte | arm  park  square  bridge | vaca  (cow) | corda  nave  figo  lume | rope  ship  fig  fire | peixe  (fish) | exame  evento  frase  capela | exam  event  phrase  chapel |
| cama  (bed) | lenda  dor  lago  lume | legend  pain  lake  fire | termo  (thermos) | alvo  sinal  pedra  carne | target  signal  stone  flesh | galinha  (chicken) | torneira  semente  martelo  detalhe | tap  seed  hammer  detail | cavalo  (horse) | ofício  azeite  placa  neve | craft  olive oil  board  snow |
| cadeira  (chair) | farinha  nave  senado  teste | flour  ship  senate  test | relógio  (watch) | tabaco  certame  medalha  árvore | tobacco  event  medal  tree | águia  (eagle) | cifra  creche  hélio  cabaz | digit  nursery  helium  basket | rato  (rat) | molho  iate  faca  mente | sauce  yacht  knife  mind |
| carteira  (wallet) | revolta  gravidade  tornado  recital | revolt  gravity  tornado  recital | prato  (plate) | circo  iate  espada  cruz | circus  yacht  sword  cross | ovelha  (sheep) | medula  cabine  soneto  arame | marrow  cabin  sonnet  wire | dinossauro  (dinosaur) | telescópio  chocolate  ferramenta  extremidade | telescope  chocolate  tool  limb |
| bicicleta  (bicycle) | camisola  tempestade  catálogo  passaporte | sweater  storm  catalog  passport | copo  (glass) | dedo  poste  horta  peste | finger  post  vegetable  garden  plague | cabra  (goat) | túnica  tarte  astro  capote | robe  pie  star  cloak | pássaro  (bird) | ginásio  vinagre  lâmpada  manchete | gymnasium  vinegar  lamp  headline |
| flor  (flower) | maré  dieta  arroz  ombro | tide  diet  rice  shoulder | globo  (globe) | joelho  tomate  raiva  raíz | knee  tomato  rage  root | raposa  (fox) | orelha  foice  terraço  ciclone | ear  scythe  terrace  cyclone | polvo  (octopus) | crânio  bosque  tecla  fronte | skull  grove  key  forehead |
| garrafa  (bottle) | fogueira  validade  terraço  tanque | fire pit  expiration  terrace  tank | queijo  (cheese) | sismo  baile  relva  elite | earthquake  ball, dance  grass  elite | aranha  (spider) | cevada  estante  gelado  dique | barley  bookcase  ice cream  dike | crocodilo  (crocodile) | relâmpago  detergente  azeitona  tangente | lightning  detergent  olive  tangent |
| gaveta  (drawer) | prenda  claque  casino  arame | gift  fan group  casino  wire | casaco  (jacket) | moinho  apetite  barriga  praxe | mill  appetite  belly  hazing | mosca  (fly) | panela  couve  lábio  truque | pan  cabbage  lip  trick | sapo  (toad) | trigo  pote  rolha  vide | wheat  bowl  cork  vine branch |
| lata  (can) | mola  cave  selo  dente | clothespin  basement  seal  tooth | tapete  (rug) | bigode  útero  morgue  galáxia | moustache  uterus  morgue  galaxy | bruxa  (witch) | toalha  sande  lenço  cofre | towel  sandwich  scarf  safe box | papagaio  (parrot) | antídoto  sabonete  amêndoa  gabardine | antidote  soap  almond  gabardine |
| gravata  (tie) | cebola  estante  centeio  bosque | onion  bookcase  rye  grove | berço  (cradle) | crânio  farol  tecla  foice | skull  lighthouse  key  scythe | lagosta  (lobster) | bússola  falange  charuto  abacate | compass  phalanx  cigar  avocado | golfinho  (dolphin) | marmelo  peluche  amêijoa  esfinge | quince  plush  clam  sphinx |
| toalha  (towel) | âncora  couve  átomo  cabaz | anchor  cabbage  atom  basket | frigorífico  (refrigerator) | microscópio  transplante  recompensa  tuberculose | microscope  transplant  reward  tuberculosis | tartaruga  (turtle) | beterraba  avalanche  candeeiro  esparguete | beet  avalanche  lamp  spaghetti | camelo  (camel) | fungo  frete  saliva  acidez | fungus  freight  saliva  acidity, sourness |
| chaminé  (chimney) | entorse  lágrima  foguete  teclado | sprain  tear  rocket  keyboard | armário  (closet) | veneno  vinagre  lanterna  diálise | poison  vinegar  flashlight  dialysis | avestruz  (ostrich) | maionese  gelatina  enxame  remorso | mayonnaise  jelly  swarm  remorse | mosquito  (mosquito) | azevinho  disfarce  alcatifa  planície | holly  disguise  carpet  plain |
| torneira  (tap) | almofada  dinamite  carimbo  barrete | pillow  dynamite  stamp  cap | colete  (vest) | ventre  frasco  lápide  fábula | abdomen  bottle  tombstone  fable | borboleta  (butterfly) | mangueira  obesidade  eucalipto  envelope | hose  obesity  eucalyptus  envelope | caranguejo  (crab) | termómetro  piquenique  trincheira  sanduíche | thermometer  picnic  trench  sandwich |
| mochila  (backpack) | alergia  cabine  cotovelo  ciclone | allergy  cabin  elbow  cyclone | sapato  (shoe) | azulejo  cacete  botija  prótese | tile  baguette  cylinder  prosthesis | joaninha  (ladybug) | fotocópia  disquete  isqueiro  rabanete | photocopy  floppy disk  lighter  radish | lagarto  (lizard) | tempero  chicote  camélia  maquete | seasoning  whip  camellia  model |
| chávena  (cup) | bússola  sirene  isqueiro  diamante | compass  siren  lighter  diamond | aquário  (aquarium) | tempero  disfarce  pulseira  hélice | seasoning  disguise  bracelet  propeller | abelha  (bee) | peruca  hélice  estojo  quiosque | wig  propeller  case  kiosk | veado  (deer) | palito  cone  pomada  grade | toothpick  cone  ointment  grille |
| vassoura  (broom) | esmeralda  maionese  parafuso  fiambre | emerald  mayonnaise  screw  ham | vaso  (vase) | fungo  pote  telha  trave | fungus  bowl  roof tile  beam | coruja | acácia  elipse  vinilo  xarope | acacia  ellipse  vinyl  syrup | morcego  (bat) | fósforo  cabide  cereja  epiderme | match  hanger  cherry  epidermis |
| rolha  (cork) | laca  lente  mofo  cone | lacquer  lens  mold  cone | candeeiro  (lamp) | brinquedo  envelope  azeitona  sanduíche | toy  envelope  olive  sandwich | minhoca | peçonha  laringe  topázio  foguete | venom  larynx  topaz  rocket | hipopótamo  (hippopotamus) | candelabro  espinafre  cambalhota  salinidade | chandelier  spinach  back flip  salinity |
| sanita  (toilet) | geleia  tarte  baloiço  cabide | jam  pie  swing  hanger | fósforo  (match) | cogumelo  chicote  abóbora  esfinge | mushroom  whip  pumpkin  sphinx | girafa | bolota  virose  caroço  colete | acorn  virosis  pit  vest | pelicano  (pelicano) | biscoito  xilofone  espátula  limousine | cookie  xylophone  spatula  limousine |
| cesta  (basket) | borga  grade  feno  vime | spree (fun)  grille  hay  wicker | cinzeiro  (astray) | morango  alfinete  gelatina  laringe | strawberry  pin  gelatine  larynx | zebra | sarna  coice  beiço  pevide | scabies  kick  lip  fruit seed | esquilo  (squirrel) | repolho  barrote  alheira  raquete | cabbage  beam  type of sausage  racket |

*Note*. From top to bottom of the distractors columns: gender congruent nouns with the same transparency category of that of the target noun, gender congruent nouns with a different transparency category, gender incongruent nouns with the same transparency category, gender incongruent nouns with a different transparency category.

Table A2

*Target and distractor nouns for Experiment 1 (third condition of animacy presence)*

| **Feminine Targets** | **Distractors** | | **Masculine Targets** | **Distractors** | |
| --- | --- | --- | --- | --- | --- |
| bicicleta  (bicycle) | camisola  tempestade  catálogo  passaporte | sweater  storm  catalog  passport | peixe  (fish) | exame  evento  frase  capela | exam  event  phrase  chapel |
| vaca  (cow) | corda  nave  figo  lume | rope  ship  fig  fire | globo  (globe) | joelho  tomate  raiva  raíz | knee  tomato  rage  root |
| toalha  (towel) | âncora  couve  átomo  cabaz | anchor  cabbage  atom  basket | cavalo  (horse) | ofício  azeite  placa  neve | craft  olive oil  board  snow |
| águia  (eagle) | cifra  creche  hélio  duche | digit  nursery  helium  basket | tapete  (rug) | bigode  útero  morgue  galáxia | moustache  uterus  morgue  galaxy |
| chaminé  (chimney) | entorse  lágrima  foguete  teclado | sprain  tear  rocket  keyboard | polvo  (octopus) | bagaço  colete  fralda  fronte | skull  vest  diaper  forehead |
| aranha  (spider) | cevada  estante  gelado  dique | barley  bookcase  ice cream  dike | berço  (cradle) | crânio  farol  tecla  coice | skull  lighthouse  key  kick |
| torneira  (tap) | almofada  dinamite  carimbo  barrete | pillow  dynamite  stamp  cap | crocodilo  (crocodile) | relâmpago  detergente  gabardina  tangente | lightning  detergent  raincoat  tangent |
| mosca  (fly) | panela  sande  lábio  truque | pan  sandwich  scarf  safe box | frigorífico  (refrigerator) | microscópio  transplante  recompensa  tuberculose | microscope  transplant  reward  tuberculosis |
| mochila  (backpack) | alergia  cabine  cotovelo  ciclone | allergy  cabin  elbow  cyclone | golfinho  (dolphin) | marmelo  peluche  castanha  esfinge | quince  plush  chestnut  sphinx |
| tartaruga  (turtle) | beterraba  avalanche  guardanapo  esparguete | beet  avalanche  napkin  spaghetti | sapato  (shoe) | azulejo  cacete  botija  prótese | tile  baguette  cylinder  prosthesis |
| vassoura  (broom) | esmeralda  cicatriz  parafuso  fiambre | emerald  scar  screw  ham | camelo  (camel) | fungo  frete  saliva  acidez | fungus  freight  saliva  acidity, sourness |
| avestruz  (ostrich) | maionese  gelatina  enxame  remorso | mayonnaise  jelly  swarm  remorse | candeeiro  (lamp) | brinquedo  envelope  azeitona  sanduíche | toy  envelope  olive  sandwich |
| rolha  (cork) | laca  lente  mofo  cone | lacquer  lens  mold  cone | morcego  (bat) | semáforo  iogurte  cereja  epiderme | traffic light  yoghurt  cherry  epidermis |
| borboleta  (butterfly) | mangueira  obesidade  eucalipto  trombone | hose  obesity  eucalyptus  trombone | fósforo  (match) | cogumelo  chicote  abóbora  alface | mushroom  whip  pumpkin  lettuce |
| sanita  (toilet) | geleia  tarte  baloiço  cabide | jam  pie  swing  hanger | rato  (rat) | molho  poste  faca  mente | sauce  post  knife  mind |
| zebra  (zebra) | sarna  foice  beiço  legume | scabies  kick  lip  fruit seed | cinzeiro  (ashtray) | morango  alfinete  bolacha  laringe | strawberry  pin  cookie  larynx |
| chávena  (cup) | bússola  sirene  isqueiro  diamante | compass  siren  lighter  diamond | hipopótamo  (hippopotamus) | candelabro  espinafre  cambalhota  salinidade | chandelier  spinach  back flip  salinity |
| abelha  (bee) | peruca  lactose  estojo  quiosque | wig  propeller  case  kiosk | prato  (plate) | circo  iate  espada  cruz | circus  yacht  sword  cross |
| gravata  (tie) | cebola  claque  centeio  bosque | onion  fan group  rye  grove | papagaio  (parrot) | antídoto  sabonete  amêndoa  gabardine | antidote  soap  almond  gabardine |
| galinha  (chicken) | canela  semente  martelo  detalhe | cinnamon  seed  hammer  detail | queijo  (cheese) | sismo  baile  relva  elite | earthquake  ball, dance  grass  elite |
| carteira  (wallet) | revolta  gravidade  tornado  recital | revolt  gravity  tornado  recital | caranguejo  (crab) | termómetro  piquenique  trincheira  amigdalite | thermometer  picnic  trench  tonsillitis |
| raposa  (fox) | orelha  vitrine  terraço  alicate | ear  glass cabinet  terrace  cyclone | casaco  (jacket) | moinho  apetite  barriga  praxe | mill  appetite  belly  hazing |
| lata  (can) | mola  cave  selo  dente | clothespin  basement  seal  tooth | dinossauro  (dinosaur) | telescópio  chocolate  ferramenta  extremidade | telescope  chocolate  tool  limb |
| girafa  (giraffe) | bolota  virose  caroço  abacate | acorn  virosis  pit  avocado | aquário  (aquarium) | tempero  disfarce  pulseira  hélice | seasoning  disguise  bracelet  propeller |

*Note*. From top to bottom of the distractors columns: gender congruent nouns with the same transparency category of that of the target noun, gender congruent nouns with a different transparency category, gender incongruent nouns with the same transparency category, gender incongruent nouns with a different transparency category.

Table A3

*Target and distractor nouns for Experiment 2*

| **Feminine Targets** | **Distractors** | | **Masculine Targets** | **Distractors** | |
| --- | --- | --- | --- | --- | --- |
| bicicleta  (bicycle) | camisola  tempestade  catálogo  passaporte | sweater  storm  catalog  passport | peixe  (fish) | cartaz  evento  frase  capela | exam  event  phrase  chapel |
| vaca  (cow) | corda  nave  figo  lume | rope  ship  fig  fire | globo  (globe) | joelho  tomate  raiva  raíz | knee  tomato  rage  root |
| toalha  (towel) | âncora  couve  granito  cabaz | anchor  cabbage  granite  basket | cavalo  (horse) | ofício  azeite  placa  neve | craft  olive oil  board  snow |
| águia  (eagle) | cifra  creche  hélio  duche | digit  nursery  helium  shower | tapete  (rug) | vapor  útero  foice  pimenta | vapour, steam  uterus  scythe  pepper |
| chaminé  (chimney) | entorse  lágrima  bigode  teclado | sprain  tear  moustache  keyboard | relógio  (watch) | tabaco  xadrez  manteiga  humidade | tobacco  chess  butter  humidity |
| aranha  (spider) | cevada  estante  gelado  dique | barley  bookcase  ice cream  dike | berço  (cradle) | crânio  farol  tecla  coice | skull  lighthouse  key  kick |
| torneira  (tap) | almofada  dinamite  carimbo  barrete | pillow  dynamite  stamp  cap | crocodilo  (crocodile) | relâmpago  detergente  gabardina  tangente | lightning  detergent  raincoat  tangent |
| flor  (flower) | maré  dieta  arroz  ombro | tide  diet  rice  shoulder | frigorífico  (refrigerator) | contentor  transplante  recompensa  tuberculose | dumpster  transplant  reward  tuberculosis |
| mochila  (backpack) | alergia  cabine  rebanho  ciclone | allergy  cabin  flock, herd  cyclone | armário  (closet) | veneno  vinagre  lanterna  diálise | poison  vinegar  flashlight  dialysis |
| tartaruga  (turtle) | beterraba  avalanche  chouriço  esparguete | beet  avalanche  smoked sausage  spaghetti | sapato  (shoe) | rótulo  cacete  botija  prótese | tile  baguette  cylinder  prosthesis |
| vassoura  (broom) | esmeralda  cicatriz  intestino  fiambre | emerald  scar  intestine  ham | copo  (glass) | dedo  poste  horta  peste | finger  post  vegetable garden  plague |
| avestruz  (ostrich) | maionese  gelatina  enxame  remorso | mayonnaise  jelly  swarm  remorse | candeeiro  (lamp) | brinquedo  envelope  basílica  sanduíche | toy  envelope  basilica  sandwich |
| rolha  (cork) | laca  lente  prado  cone | lacquer  lens  meadow  cone | colete  (vest) | ventre  frasco  lápide  fábula | abdomen  bottle  tomstone  fable |
| cadeira  (chair) | farinha  semente  senado  teste | flour  seed  senate  test | fósforo  (match) | recibo  chicote  abóbora  alface | receipt  whip  pumpkin  lettuce |
| sanita  (toilet) | geleia  tarte  lábio  cabide | jam  pie  lip  hanger | rato  (rat) | molho  pente  palha  mente | sauce  comb  straw  mind |
| cama  (bed) | lenda  dor  lago  gene | legend  pain  lake  gene | cinzeiro  (ashtray) | morango  revólver  bolacha  laringe | strawberry  gun  cookie  larynx |
| chávena  (cup) | bússola  sirene  isqueiro  diamante | compass  siren  lighter  diamond | hipopótamo  (hippopotamus) | candelabro  espinafre  cambalhota  salinidade | chandelier  spinach  back flip  salinity |
| cesta  (basket) | borga  grade  feno  vime | spree (fun)  grille  hay  wicker | prato  (plate) | circo  iate  espada  cruz | circus  yacht  sword  cross |
| gravata  (tie) | cebola  claque  centeio  bosque | onion  fan group  rye  grove | vaso  (vase) | fungo  pote  telha  trave | fungus  bowl  roof tile  beam |
| gaveta  (drawer) | prenda  estirpe  casino  arame | gift  strain  casino  wire | queijo  (cheese) | sismo  baile  relva  elite | earthquake  ball, dance  grass  elite |
| carteira  (wallet) | revolta  gravidade  tornado  recital | revolt  gravity  tornado  recital | termo  (thermos) | navio  exame  loja  carne | ship  exam  store  flesh |
| raposa  (fox) | orelha  vitrine  estojo  alicate | ear  glass cabinet  case  pliers | casaco  (jacket) | moinho  apetite  barriga  praxe | mill  appetite  belly  hazing |
| lata  (can) | mola  cave  selo  dente | clothespin  basement  seal  tooth | dinossauro  (dinosaur) | telescópio  chocolate  ferramenta  extremidade | telescope  chocolate  tool  limb |
| garrafa  (bottle) | fogueira  validade  terraço  tanque | fire pit  expiration  terrace  tank | aquário  (aquarium) | tempero  vector  pulseira  hélice | seasoning  vector  bracelet  propeller |

*Note*. From top to bottom of the distractors columns: gender congruent nouns with the same transparency category of that of the target noun, gender congruent nouns with a different transparency category, gender incongruent nouns with the same transparency category, gender incongruent nouns with a different transparency category.

Table A4

*Mean incorrect responses and standard errors (SE) for each condition in Experiment 1*

| **Animacy** | **Gender** | **Congruency** | **Mean** | ***SE*** |
| --- | --- | --- | --- | --- |
| 0% | Masculine | Incongruent | 0.11 | 0.02 |
|  |  | Congruent | 0.08 | 0.01 |
|  | Feminine | Incongruent | 0.06 | 0.01 |
|  |  | Congruent | 0.09 | 0.02 |
|  |  |  |  |  |
| 50% | Masculine | Incongruent | 0.04 | 0.01 |
|  |  | Congruent | 0.04 | 0.01 |
|  | Feminine | Incongruent | 0.07 | 0.01 |
|  |  | Congruent | 0.04 | 0.01 |
|  |  |  |  |  |
| 100% | Masculine | Incongruent | 0.09 | 0.02 |
|  |  | Congruent | 0.08 | 0.01 |
|  | Feminine | Incongruent | 0.13 | 0.02 |
|  |  | Congruent | 0.12 | 0.02 |
|  |  |  |  |  |

*Note*. Animacy stands for factor “Animacy presence”, Gender stands for factor “Target gender”, Congruency stands for factor “Gender congruency”.

Table A5

*Mean incorrect responses and standard errors (SE) for each condition in Experiment 2*

| **Gender** | **Congruency** | **Mean** | ***SE*** |
| --- | --- | --- | --- |
| Masculine | Incongruent | 0.09 | 0.01 |
|  | Congruent | 0.08 | 0.01 |
| Feminine | Incongruent | 0.07 | 0.01 |
|  | Congruent | 0.06 | 0.01 |
|  |  |  |  |

*Note*. Gender stands for factor “Target gender”, Congruency stands for factor “Gender congruency”.
